# A quality improvement initiative to improve developmental screening in a high-risk follow-up clinic in western India

**DOI:** 10.3389/fped.2025.1648694

**Published:** 2025-10-30

**Authors:** Arshdeep Kaur, Arpit Sohane, Priyadarshini Virupaxi Chougula, Nandini Malshe, Pradeep Suryawanshi, Suprabha Patnaik

**Affiliations:** Department of Neonatology, Bharati Vidyapeeth Deemed University Medical College and Hospital, Pune, India

**Keywords:** Ages and Stages Questionnaire-3, ASQ-3, development screening, quality improvement, high-risk follow-up clinic

## Abstract

**Introduction:**

Early childhood is a uniquely sensitive period for developing cognitive ability, language, social and motor development. Any disruption in the typical progression within these areas may result in developmental delays if not intervened timely, potentially causing various morbidities in a child and adversely affecting the quality of life for both the child and their family. Therefore, it is essential to conduct developmental screenings at regular recommended intervals.

**Methods:**

This single-center quality improvement (QI) study was conducted in a tertiary care center with a level 3B neonatal intensive care unit, running a high-risk follow-up clinic. We aimed to improve the developmental screening in children (≤2 years) visiting the high-risk follow-up clinic, from the current 46.1% to ≥75% within three months. A quality improvement team conducted a root cause analysis for low screening rate, which led to the planning of several interventions. These included ensuring the consistent availability of Ages and Stages Questionnaire-3 (ASQ-3) screening forms in both regional and official languages, establishing a dedicated ASQ-3 counter, and conducting reinforcement and training sessions for interns and junior residents. Data was logged in the clinic record book and analyzed with statistical process control chart (P chart) and a run chart.

**Results:**

This QI project commenced in October 2023 and is still operational in a sustainable phase. During the study period, out of 1,368 eligible children, 1,259 (92%) underwent developmental screening using the ASQ-3 screening tool (*process indicator*). Developmental delay was detected in 196 children (15.5%), who were referred for early intervention (*outcome indicator*). Process was under statistical control, as evidenced by P charts.

**Conclusion:**

This QI project improved the clinic's ASQ-3 performance rate, thereby helping more children with developmental delay obtain early intervention.

## Introduction

Early childhood is a sensitive period for developing cognitive ability, language, social, and motor development. If not addressed promptly, deviation from normal development in any of these domains may lead to developmental delay. Therefore, early identification and appropriate intervention are critical for optimizing language, cognitive, motor, socioemotional development and educational success (1–3). Before the age of 2 years, due to neuronal plasticity, children with developmental delay can attain maximum benefit if they receive the proper intervention at the right time (4–6).

Globally, there remains a substantial gap between identification of developmental delays and receipt of early intervention: In United States (US) only an estimated 10% of children with delays receive intervention by 24 months, despite 13% being eligible (7). In India, this gap is even more pronounced, a study from a Northern Indian Child Development and Early Intervention Clinic found that 37.1% of children with neurodevelopmental disabilities were referred at 3 years or older, reflecting widespread delayed referral leading to loss of opportunity for early intervention (8). In another study, the median age for parental concern among children with developmental disabilities, excluding autism spectrum disorder was 7 months, yet the median age of referral to rehabilitation services was 13 months, indicating significant delays in early access to developmental services (9). This striking disparity highlights the urgent need for routine developmental screening models following standard and structured screening recomendations.

The American Academy of Pediatrics recommends developmental screening during regular well-child visits at 9, 18, and 24 or 30 months (10). The Indian Academy of Pediatrics (IAP) recommends routine developmental surveillance for all children at every immunization visit until two years of age, using milestone-specific inquiries appropriate for the child's current age. Developmental screening using standardized tools is advised at 9–12 months, 18–24 months, and again at school entry with additional surveillance and screening for high risk neonates at 4–6 months of age, then yearly till 5 years and once at school entry (11).

The Ages and Stages Questionnaire is a parent completed developmental screening tool, designed and developed by J. Squires and D. Bricker, at the University of Oregon (12–15). ASQ-3 has been adapted and validated in multiple countries—including Australia, Brazil, Canada, China, Ecuador, Ghana, India, Iran, Turkey, and others—demonstrating its cross-cultural utility (16). ASQ-3 is validated in both Marathi and Hindi Indian languages (17–19). Though we had been using the Ages and Stages Questionnaire-3 (ASQ-3) as a screening tool in our high-risk follow-up clinic, there was inconsistency in the practice. Probable reasons were a busy clinic and a lack of streamlining in the screening process. Realising the scope of improvement in this aspect, this quality improvement (QI) project was initiated. We formed a QI team and performed root cause analysis for inconsistency in development screening. The study's primary aim was to improve the rate of developmental screening using ASQ-3, in children from 2 months to ≤2 of years age visiting the high-risk clinic for follow-up, from the baseline of 46.1% to ≥75% within three months period. ASQ-3 is used because it is well validated and, being a parent-based development reporting tool, it becomes useful for parents to discuss any concerns that they may have regarding their child's development with a healthcare professional in a structured manner (20).

## Methods

### Study design

This was a single-center QI study.

### Study settings

This QI initiative was conducted in a tertiary care teaching hospital in western India. The hospital has a 60-bedded, level 3B neonatal intensive care unit (NICU) care facility with 1,500 admissions annually, and a clinic for high-risk follow-up after discharge from the NICU. In our high-risk follow-up clinic around 5,000 children visit annually, with the majority being under 2 years of age. The clinic is staffed with two senior neonatologists, two to four junior residents, one to two senior residents, and a trainee neonatologist and well-organized telephonic visit reminder system managed by the clinic coordinator is in place.

### Team formation and process mapping

**Figure 1:**
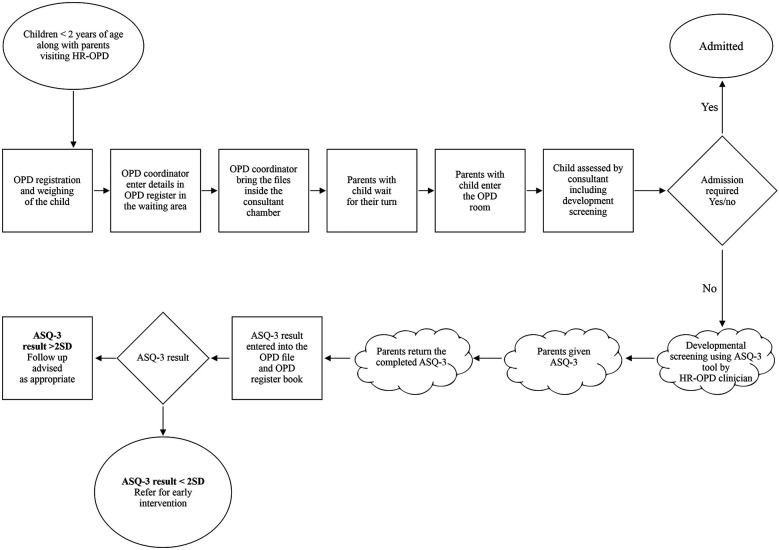
Process flow mapping of high-risk follow-up clinic operations and ASQ-3 based development screening.

**Figure 2:**
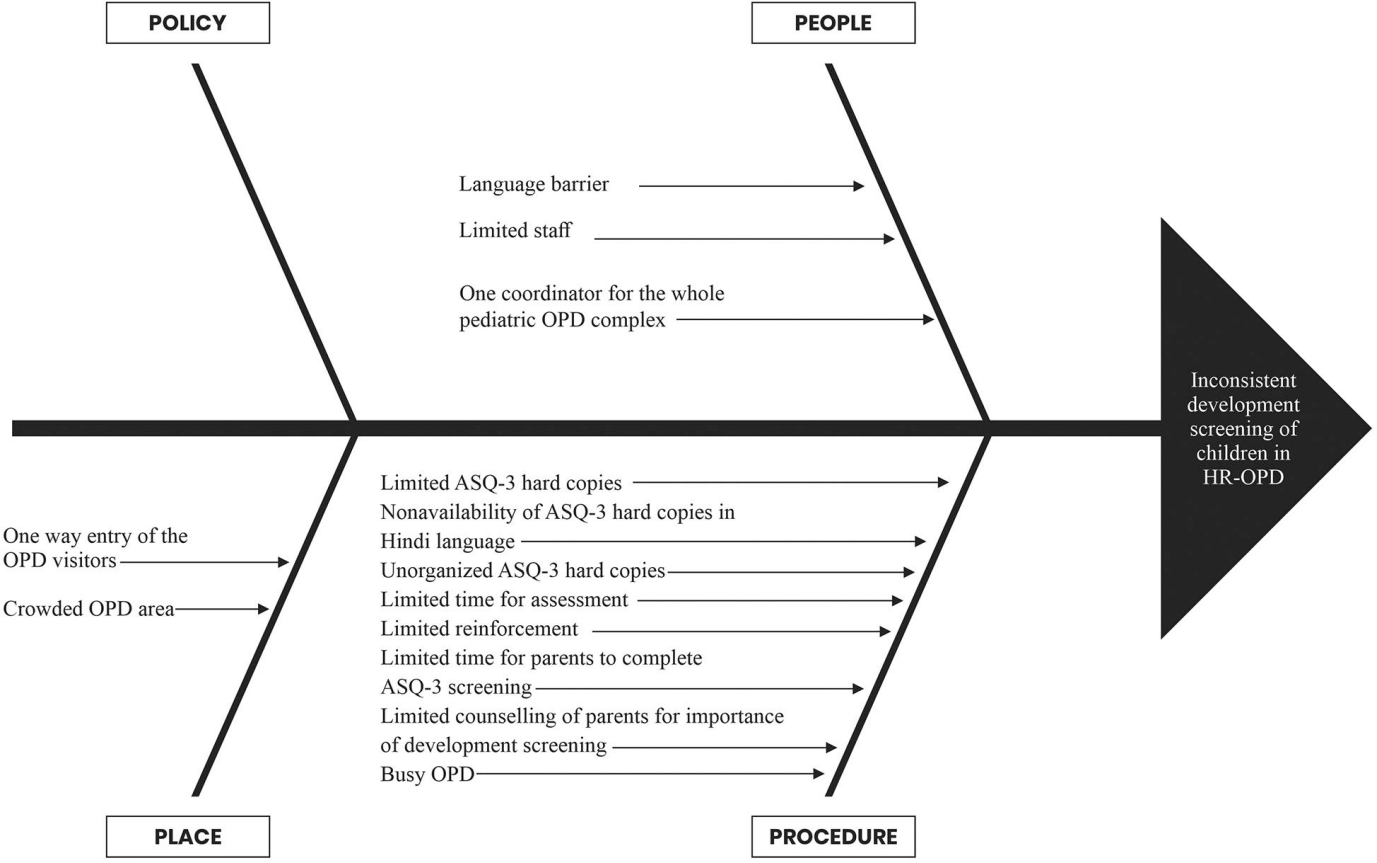
Fishbone analysis to identify the root causes of inconsistency in development screening in HR-OPD.

The QI team comprised of senior neonatologists, senior residents, junior residents, intern doctors, clinic nursing staff, and a coordinator. The team looked at the current process flow (Figure 1) and performed a root cause analysis to identify the underlying issues contributing to the suboptimal performance in developmental screening, utilizing fishbone analysis (Figure 2). The team agreed upon process and outcome indicators, and defined eligibility criteria as all children between 2 and 24 months of age (corrected gestational age if born preterm) visiting the clinic. Sick children requiring admission were excluded. Following team formation and process mapping, a team leader was appointed to monitor the QI project, address any shortcoming, and troubleshoot problems. The flow of the QI process was consistently monitored through regular team meetings.

### Materials

#### ASQ-3 screening tool

ASQ-3 assesses five developmental domains – fine motor, gross motor, communication, problem solving, and personal-social, nine open-ended questions and six close-ended questions for each domain, consisting of total 30 questions that can be answered “yes,” “sometimes,” or “not yet”, for scores of 10, 5, and 0, respectively. For any domain, a total score that is two standard deviations (SD) below the population mean indicates developmental delay, and with those between 1 and 2 SD below the population mean are considered in monitoring zone as specified in the ASQ-3 technical manual (14, 15). Marathi and Hindi version of ASQ-3 which has been previously validated in India, was utilized for this QI study (17–19).

#### ASQ-3 screening in high-risk follow-up clinic

Parents received printed age specific ASQ-3 forms in Marathi or Hindi language, during their child's visit in high-risk follow-up clinic. For the assessment, locally available materials, like pencils or crayons were supplied to mark the questionnaire. Developmentally appropriate toys and items were also provided to facilitate the screening process. The support was provided by the interns and the junior residents (for language, interpretation, clarification etc.) which ensured accurate completion of ASQ-3 screening. The screening was conducted initially at the time of consultation, which was later done prior to the clinician's evaluation of the child. The ASQ-3 score was evaluated by the consultant and documented it in the OPD records. This ensured a controlled environment and seamless integration into the clinic flow, which was achieved through multiple PDSA cycles as decribed below.

#### Strategy

We followed the World Health Organization model for quality improvement, “POCQI-point of care quality improvement”. Several Plan-Do-Study-Act (PDSA) cycles were implemented as components of the QI initiative throughout the study. Departmental meetings and group discussions took place at the onset of each PDSA cycle. The long-term sustainability of the intervention was ensured through ongoing reinforcement and educational sessions. Following nine PDSA cycles were conducted:

##### PDSA cycle 1: ensuring regular availability of printed ASQ-3 forms

The team proposed that the primary intervention should ensure regular availability of hard copies of ASQ-3 for all age groups. One senior resident was assigned this task to ensure the availability of these copies for the subsequent three clinic days. We implemented this change for one week (3 clinic days). We observed that this change in practice was possible for the senior resident without facing any challenge, and this proposed idea worked well. Hence, it was adopted.

##### PDSA cycle 2: organizing ASQ-3 forms by age group

During the testing phase of PDSA cycle 1, it became evident that ensuring adequate ASQ-3 form availability would not resolve the entire problem. Before the commencement of this QI project, ASQ-3 forms for different age groups were kept cluttered together in a single folder, which made it difficult for the team members to find the age-appropriate questionnaire on time amidst the busy clinic. This led to inconsistency in developmental screening. To address this issue, the team proposed organizing the questionnaire in distinct folders labelled by age (in months), facilitating easy and prompt distribution of ASQ-3 forms to the parents. The proposal was followed for a week and found feasible. The strategy saved time, which was utilised for counselling and assisting the parents in completing the questionnaire, leading us to formally adopt this change.

##### PDSA cycle 3: making available multilingual ASQ-3 forms

The city where this QI project was conducted caters to a linguistically diverse population. Consequently, many parents cannot write, read, or speak the state's local language. Before implementing this QI project, ASQ-3 was available in only the local language (Marathi). After realizing this problem, questionnaire was made available in official language of India (Hindi). For those who could not understand the local or official language, a junior residents interviewed the parents and filled in their evaluation responses. This change initiative improved the ASQ-3 screening coverage in the clinic, leading us to adopt this approach.

##### PDSA cycle 4: involvement of intern doctors

The team decided to engage the interns posted in the Neonatology department to improve the developmental screening coverage. Introductory lectures on the significance of developmental screening in the clinic and training sessions on effective counselling of parents, along with guiding them to complete the ASQ-3 screening, were conducted by the senior neonatology faculty and/or senior resident. This change in practice was observed for the next week, and it resulted in improving ASQ-3 screening coverage. Hence, the idea was adapted.

##### PDSA cycle 5: establishment of an “ASQ-3 counter” in the clinic

The team realised that it was difficult to counsel the parents and engage them to complete the ASQ-3 screening during the working operations of the high-risk follow-up clinic. Hence, we devised the idea to establish a designated area within the clinic for ASQ-3 screening. The clinic coordinator would direct the parents to this “ASQ-3 Counter” where an intern or junior resident would counsel them regarding the importance of ASQ-3 developmental screening and help them in screening. This saved time for both parents and the consultant neonatologist, as parents would undergo ASQ-3 screening before visiting the consultant, who could evaluate the ASQ-3 scores during the meeting with the parents. We tested this change for one week, and we adopted the change since it yielded positive results.

##### PDSA cycle 6: relocation of ASQ-3 counter

We observed that even after getting appropriate directions from the clinic coordinator, some parents reported that they could not locate the ASQ-3 Counter in the clinic premises and missed the developmental screening. The team therefore relocated the counter to the front of the clinic entrance, ensuring better visibility and accessibility. This idea change was tested for another week and as it improved our ASQ-3 coverage, it was adopted permanently.

##### PDSA cycle 7: senior resident involvement and adequate training of newly posted junior residents

In the 12th week of the project, a batch of junior residents had to be relieved from clinic duty for other tasks, resulting in reduced human resources, and the screening rate declined to 78.5% from 83.3%. The team therefore decided to involve senior residents for counselling and assisting the parents with ASQ-3 screening. After one week of testing this idea, we had to abandon this idea because senior residents were essential for effectively running the clinic, and they struggled to manage both responsibilities simultaneously. In due course, a new batch of junior residents could be assigned for clinic duty, and we focused on their adequate training.

##### PDSA cycle 8: adequate training of newly posted junior residents and intern doctors

During the 17th week of the project, we observed a sharp decline in the ASQ-3 performance to 68.7%. We attributed it to the rotational posting of interns and junior residents. The freshly joined batch of junior residents and intern doctors were not adequately trained for counselling and helping the parents to complete the ASQ-3 screening. Education and training sessions for newly joined interns and junior residents were therefore organized by the senior neonatologist and/or by the senior residents via seminars and video lectures. ASQ-3 coverage increased to 75.6% and 91.6% in the following two consecutive weeks. Hence, we incorporated the training of a new batch of junior doctors and intern doctors in our practice.

##### PDSA cycle 9: withholding files until completion of ASQ-3 based developmental screening

It was observed that despite collecting the questionnaire from the designated ASQ-3 counter, some parents did not complete the developmental screening before their meeting with the consultant neonatologist. To ensure completion of the ASQ-3 screening, the team recommended that patient files be withheld until the screening was completed and the scores documented in both the patient's file and the clinic record book.

### Ongoing education

Weekly educational sessions are being conducted to educate new rotating intern doctors and junior residents about the significance of developmental screening and early interventions through seminars and video lectures, to ensure the project sustainability.

### Data collection

Two independent observers manually collected the baseline data to determine the development screening rate over the past six months prior to the commencement of this project (April-September 2023), following which, a clinic record book was used to log patient visit and screening outcome to monitor screening rates. A senior resident compiled details to share during biweekly QI team meetings. The reasons for not conducting ASQ-3 screening were identified and addressed in a timely manner. The process flow of this QI initiative was consistently monitored through the team meetings and monthly departmental audits, led by a senior neonatologist, to discuss issues, identify root causes, and implement corrective measures.

### Measures

The *process indicator* was defined as the percentage of eligible children being screened using the ASQ-3, with the numerator being the number of eligible children screened and the denominator representing the total number of eligible children multiplied by 100.

The *outcome indicator* was defined as the percentage of developmental delays detected as per ASQ-3, the numerator being the number of children detected as developmental delay as per ASQ-3 screening, and the denominator being the number of eligible children screened multiplied by 100.

### Analysis

**Figure 3:**
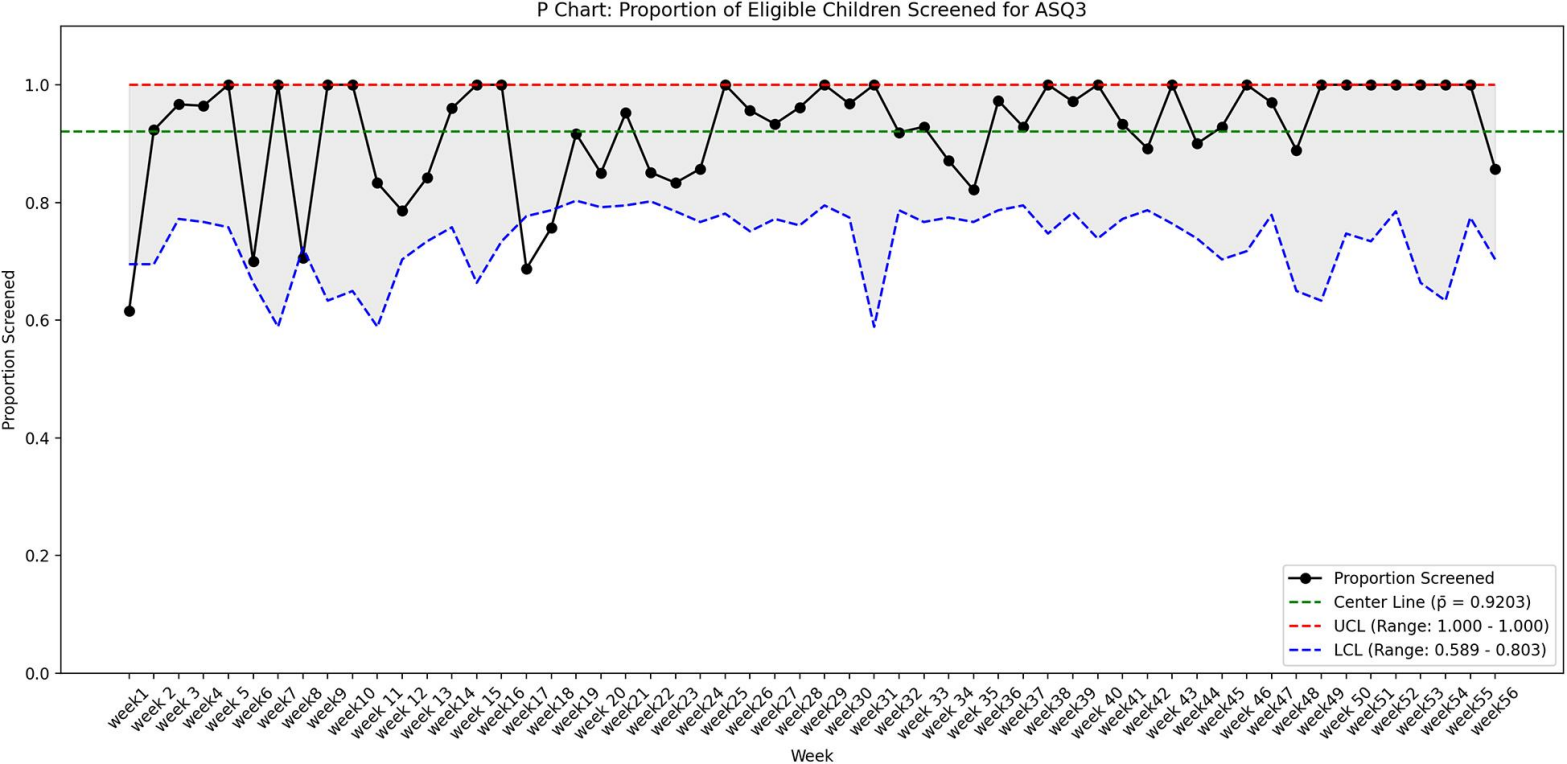
The P-chart showing the proportion of eligible children screened for ASQ-3 over 56 weeks, with a center line (mean) at 92% (*p¯* = 0.9203). The upper control limit (UCL) is capped at 1.0 (100%), while the lower control limit (LCL) varies between 58.9% and 80.3%, reflecting weekly sample size fluctuations.

**Figure 4:**
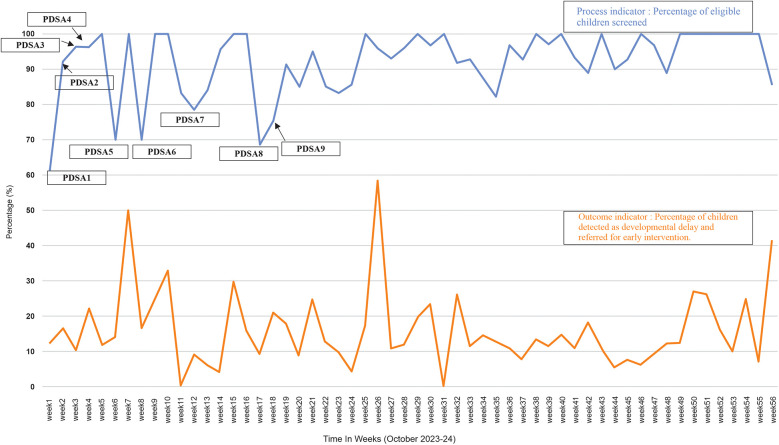
Run chart showing project indicators along with the PDSA cycles. The blue line indicates the percentage of eligible children screened and the PDSA cycles carried out at different times. The orange line shows the percentage of children identified with a developmental delay (<2SD) and referred for early intervention.

The process measure was interpreted by utilizing a statistical process control P chart created in R software (Figure 3) and by using run charts (Figure 4). The control limits were set at the 95th percentile (3-sigma). Using 3-sigma limits ensures that only significant deviations trigger investigations, confirming consistent implementation of the screening process over time. The outcome measure was assessed by a run chart (Figure 4). *Z* test for two proportions was utilized to compare rates of developmental screening amongst the eligible children before and after starting the QI initiative.

## Results

### Process indicator

Of the 6,279 total children, who visited the clinic during the study period (October 2023–24) 1,368 met the study's eligibility criteria and 1,259 (92%) eligible children underwent ASQ-3 developmental screening. In contrast, screening rate was 46.1% during the six months prior to the commencement of the project. *Z* test for two proportions revealed that observed difference in screening rates before and after the QI project was statistically significant (*p* < 0.001) (Table 1). The run chart, as seen in Figure 4, indicates that each subsequent PDSA cycle resulted in gradual improvements in screening rate. The P chart, shown in Figure 3, demonstrates an overall adherence center line of 0.9203. This confirms that using targeted interventions throughout the study period, the majority of weekly screening proportions fall within the control limits, demonstrating a stable and well-maintained process. There are no data points outside the control limits, implying the absence of special cause variation. Early weeks show slightly greater variability, but the trend stabilizes in subsequent weeks, indicating a steady compliance with the screening process. There is a consistent maintenance of proportions exceeding 90%, frequently reaching complete screening (100%), notably in the later weeks of the project. This reflects effective implementation, improved and sustained performance over time.

**Table 1:** Analysis of quality improvement project (Two proportion *Z*-test).

Pre QI implementation phase			Post QI implementation phase			*P* value
Eligible children	Number of children screened using ASQ-3 screening tool	Percentage of eligible children screened (%)	Eligible children	Number of children screened using ASQ-3 screening tool	Percentage of eligible children screened (%)	
382	176	46.07	1,368	1,259	92.03	<0.001

### Outcome indicator

ASQ-3 assessment showed that 196 (15.5%) children of those screened, had a developmental delay. In the 56-week QI project, run charts (Figure 4) were employed to illustrate the percentage of eligible children identified with developmental delays based on the ASQ-3 developmental screening and subsequently referred for early intervention.

### Post-PDSA period

This project is now in a sustainable phase, and the percentage of eligible children undergoing ASQ-3 based developmental screening has become one of the QI indicators of our unit.

## Discussion

### Summary

By employing successive PDSA cycles and incorporating team feedback, we increased ASQ-3 screening rates from 46.1% to 92%, with sustained improvement over time. During the course of this project, we detected developmental delay in 15.5% of children and referred them for diagnostic testing and early intervention. The focused team efforts, structured PDSA cycles, and learnings from them helped us to achieve the desired goal.

Although the ASQ-3 cut-off scores used in our study were based on American normative data, previous studies conducted in India show that culturally and linguistically adapted Marathi and Hindi versions of the ASQ-3 perform well. A study conducted in western India by Padbidri et al., ASQ-3 was administered in a linguistically validated Marathi version, demonstrating strong agreement with the original English version across all developmental domains with intraclass correlation coefficients ranging from 0.77 to 0.88 in Marathi-speaking, bilingual families (17). Juneja et al. states that ASQ-2 in Hindi has strong test characteristics for identifying developmental delay in Indian children, particularly in high-risk cases. This study exhibited a sensitivity of 83.3% and a specificity of 75.4% for detecting developmental delay when compared to Indian gold standard reference test, development assessment score for Indian infants (DASII). The senstivity for detecting developmental delay was even higher within high-risk subgroups (sensitivity 92.3%) (18). More recently, Gulati et al. validated a socio-culturally adapted ASQ-3 in a sample of 568 at-risk Indian infants, revealing excellent psychometric properties with overall sensitivity 95.9%, specificity 81.7% for detecting developmental delay (19). These findings support the validity of using Ages and Stages Questionnaire in Indian settings. However, we advocate for future research to develop empirically derived, population-specific ASQ norms for Indian children to enhance screening accuracy and cultural relevance.

### Interpretation

Developmental screening of the children is recommended at regular intervals (10, 21). While Hirai et al. reported that only 30.4% of US children aged 9–35 months received parent-completed developmental screening, while 37.1% received developmental surveillance from healthcare professionals, as reported by their parents or guardians (22). Coker et al. recently found only 59% of US pediatricians used standardized tools—citing barriers like time constraints, poor electronic health record integration, and limited referral pathways (23). LMICs face even more profound systemic challenges. A systematic review by Faruk et al. identified only a few culturally validated screening tools suitable for use in LMIC settings, underscoring a critical gap in accessible, feasible instruments (3). The developmental screening and surveillance rate continues to be low despite more than a decade of constant efforts. Quality improvement efforts are necessary to achieve universal screening and surveillance to optimize early identification and intervention, and monitor the developmental trajectories of children with developmental delays. Acknowledging this, a few QI studies have been conducted in the past. Malik et al. conducted a QI study in seven paediatric primary care centres to implement standardized developmental delay screening tools, and found that screening improved from 27% to 92%. Their PDSA cycles included regular training, ongoing coaching, technical support to the team members, and financial incentives to early intervention providers (24). The study differs from the present study in that Malik et al. included children from birth to 5 years, whereas our cohort included children from 2 months to 2 years of age. Moreover, the study of Malik et al. was multicentric, with a goal to standardize screening protocols across different centers. Ours is a single-center study, with the main goal was to strengthen the already existing standardized screening protocol. Malik et al. also included MCHAT screening for autism spectrum disorder, but this was not a part of our project.

Another multicenter QI project by Meurer et al., with >30,000 children, reported improvement in developmental delay screening with ASQ-3 from 60% to > 95% within 25 months for three age groups (25). The interventions included appointing clinic champions, training staff members about the screening process, using a standardized tool, posting electronic health record prompts, and offering financial incentives. Our project differed in that we did not use electronic health records, and incentives were not provided. Parents in the Meurer study completed the screening process via electronic media before clinic visit, which could have saved time in preventive health clinic operations. However, the authors reported that paediatricians found some questions in the ASQ-3 unclear to parents, necessitating further clarification and follow-up. In contrast, we assigned junior residents and intern doctors to help parents complete ASQ-3 screening in real time in the clinic. A Singapore-based QI project used a two-tiered developmental screening programme for children under < 3 years of age with a target of 80% developmental delay screening rate (26). Interventions included training primary care nurses, early-in-the-day clinic appointment slots, and limiting screening time to 20 min. It used PEDS, PEDS-Developmental Milestones (PEDS-DM), and ASQ-3 as screening tools. In contrast, we used only the ASQ-3. Regular training, team efforts, reinforcement, and process analysis are common interventions in most of the QI projects discussed above, emphasizing that these small efforts can significantly improve already existing protocols.

### Strengths of the study

Despite variations, attributable to numerous challenges encountered throughout the project, the screening coverage rate remained consistently above target for over 12 months. This was achieved through suitable and effective interventions, coupled with the QI team's dedication and commitment to uphold improved coverage. In addition to this, regular training sessions, effective counseling for parents, prompt and appropriate responses to any challenges encountered during the project, and regular team meetings held in a friendly and focused environment contributed to the attainment of improved coverage. Additionally, maintaining a prioritized list of roles for specific tasks enabled the team to implement interventions efficiently, while periodic review meetings promoted learning from feedback. Another key strength of our project was the utilization of a reliable database for baseline data collection, along with precise and real-time documentation of the process measures in clinic record book following the implementation of this QI project, enabling us to accurately assess the impact of our intervention on the screening rate. All these interventions emphasizes that minor interventions can lead to substantial changes in outcomes.

### Lessons and limitations

Despite our regular efforts, some parents inadvertently missed receiving the ASQ-3 printed copies, especially during the project's initial phase, prompting us to refine our approach. It became crucial for the physicians to provide effective counseling to the parents. We observed that good counseling skills significantly increased parents’ commitment to their child's developmental screening. Another limitation of the study is rotatory posting of junior residents and interns in the neonatology department, which is addressed by training them as soon as they join the department. Also, this study did not include feedback from the parents about how easy and helpful it was to complete the questionnaire and how it helped them to know about their child's development. Our follow-up cohort primarily consists of children up to 2 years of age; therefore, we initiated the project with this age group. However, as our project enters a sustainable phase, we intend to expand its scope to children beyond 2 years of age. Another limitation of this QI was the exclusive use of paper-based ASQ-3 administration. While paper forms are simple and require minimal infrastructure, they are labour-intensive, prone to manual scoring errors, and hinder streamlined data collection for longitudinal follow-up and research. Although the transition to electronic screening was beyond the scope and resources of the present study, future efforts could explore digital platforms to automate scoring, reduce human error, and support advanced research capabilities.

## Conclusion

In order to optimize long-term neurodevelopmental outcomes, developmental screening and early intervention are crucial health measures for high-risk newborns. This QI initiative helped us to increase ASQ-3 based development screening coverage in our hospital's high-risk follow-up clinic for children who had been admitted in its NICU. This has helped the children and parents with early intervention in developmental delays, emphasizing that good critical care and follow-up care go hand in hand. The team is now attempting to continue the same efforts, bring the change of ideas into departmental protocol, and sustain these developmental screening efforts. We hope that the lessons from our initiative will be valuable towards such efforts.

## Data Availability

The original contributions presented in the study are included in the article/Supplementary Material, further inquiries can be directed to the corresponding author.
